# Prevalence and Risk Factors Associated with Injurious Falls among Community-Dwelling Older Adults in Indonesia

**DOI:** 10.1155/2018/5964305

**Published:** 2018-06-03

**Authors:** Supa Pengpid, Karl Peltzer

**Affiliations:** ^1^ASEAN Institute for Health Development, Mahidol University, Salaya, Thailand; ^2^Department of Research & Innovation, University of Limpopo, Turfloop, South Africa; ^3^Department for Management of Science and Technology Development, Ton Duc Thang University, Ho Chi Minh City, Vietnam; ^4^Faculty of Pharmacy, Ton Duc Thang University, Ho Chi Minh City, Vietnam

## Abstract

**Objective:**

To assess the prevalence and health correlates of fall-related injury in a national population-based community-dwelling sample of older Indonesians.

**Methods:**

Participants were 6698 older adults, 50 years and older (median age 58.0 years, IQR=11.0, and age range of 50–101 years), who took part in in the Indonesia Family Life Survey (IFLS-5) in 2014-15. They provided information about sociodemographic, various health variables, including a falling down and received treatment history in the last two years.

**Results:**

Overall, 12.8% had one or more fall-related injuries in the past two years, 14.0% among women and 11.5% among men, 7.6% had a single fall, and 5.2% multiple fall-related injuries in the past two years. In multivariable logistic regression models, having two or more chronic conditions, urinary problems, and functional disability was independently associated with multiple fall-related injuries in the past two years in both sexes. Sex-specific risk factors were former tobacco use, having or having had a cataract, sleep disturbance, and sleep impairment in men and poorer economic background, depression symptoms, and low cognitive functioning in women.

**Conclusion:**

A significant proportion of older adults in Indonesia have fall-related injury. Several homogenous between the sexes and sex-specific risk factors for fall-related injury were identified that can help in designing fall-prevention strategies.

## 1. Introduction

Fall-related injury in older adults has been recognized as a major public health issue [1–3]. In a study among older adults (50 years and older) in six middle-income countries, the prevalence of past-year fall-related injuries was 4.0%, ranging from 6.6 % in India and 3.1% in China to 1.0 % in South Africa [4]. In older adults (60 years and older) in Singapore, the prevalence rate of past one year falls was 17.2%, of which one-third had recurrent falls [5], and among community-dwelling older adults (60 years and older) in Thailand, 18.7% reported having had one or more falls in the past six months [6]. In a local community-based study among older adults in Malaysia, the past-year prevalence of falls was 4.1% [7]. In a review on falls among older adults in Southeast Asia, Romli et al. [8] found that more research is needed from all Southeast Asian countries, including Indonesia, to get ready for the management of falls in an ageing society.

Effective fall reduction programmes need to include a comprehensive fall risk assessment and targeted interventions [9]. “Most of these falls are associated with one or more identifiable risk factors (e.g., weakness, unsteady gait, confusion, and certain medications), and research has shown that attention to these risk factors can significantly reduce rates of falling” [9, p.37]. Various risk factors for fall injuries in older adults have been identified, including sociodemographic, health status, and health behaviour variables. Sociodemographic risk factors include increasing age [5, 10–13], being female [4, 5, 14], lower socioeconomic status [14], and residing in rural areas [4, 15].

Health status risk factors for fall-related injuries among older adults may include nutritional risk [16], multimorbidity [4, 12, 13, 16] (including specific chronic conditions such as hypertension [5], stroke [14, 17, 18], and diabetes [18]), low hand grip strength [19], and poor cognitive functioning [4, 14]. Other health risk factors include functional disability, such as limitations of Activities of Daily Living (ADL) and Instrumental Activities of Daily Living (IADL) [4, 11, 20, 21], gait problems [22], inadequate standing balance [13], visual difficulties [14, 18], having cataracts [12], hearing problems [23, 24], urinary incontinence [13, 14, 18], and depression [4, 13]. Health risk behaviour variables associated with an increased risk of fall injuries may include physical inactivity [5, 14, 18, 24, 25], alcohol use [15, 25], cigarette smoking [26], obesity [12, 27], and sleeping problems [4, 28].

Governments in Southeast Asian countries, such as in Indonesia, need epidemiological data on fall-related injuries in order to successfully include falls prevention health care programming [4, 17]. In order to address this gap, the study aims to assess the prevalence and health correlates of fall-related injury in a national population-based community-dwelling sample of older Indonesians who participated in the Indonesia Family Life Survey (IFLS-5) in 2014-15.

## 2. Methods

### 2.1. Sample and Procedure

Data were analysed from the “Indonesia Family Life Survey (IFLS-5)”, a continuing demographic and health survey that began in 1993 and had since four rounds of data collection, with the fifth wave (IFLS-5) having been completed in 2015 [29]. The community survey collected data on household and individual level using a multistage stratified sampling [29]. The sampling frame of the first survey in 1993 was based on households from 321 enumeration areas (EAs) (20 households were randomly chosen from each urban EA and 30 households from each rural EA) in 13 out of 27 provinces that were selected representing 83% of the Indonesian population in 1993, more details in Strauss et al. [29]. At household level, several randomly selected members of the household were asked for detailed individual information. In the IFLS-5 6698 individuals 50 years and older were interviewed with complete fall-related injury measurements. In the IFLS-5, “the dynasty recontact rate was 92% and for the individual target households (including split off households as separate) the recontact rate was 90.5%.” [29]. Although the survey is longitudinal, we restricted our analysis to the IFLS-5 cross-sectional survey for persons 50 years and older, being the most recent national survey available assessing fall injuries. The IFLS has been approved by ethics review boards of RAND and University of Gadjah Mada in Indonesia [29]. Informed consent was attained from all respondents prior to assessments.

### 2.2. Measures

#### 2.2.1. Outcome Variable

*Fall-related injury *was assessed with the questions, “Have you fallen down in the last two years and received treatment?” and “How many have you fallen down and received treatment in the last two years?” [29]

#### 2.2.2. Exposure Variables

*Sociodemographic factor* questions included age, sex, education, and residential status. Subjective economic status was assessed the question “Please imagine a six-step ladder where on the bottom (the first step), stand the poorest people, and on the highest step (the sixth step), stand the richest people. On which [economic] step are you today?” The answers ranged from (1) poorest to (6) richest [29].

*Anthropometric Measurements.* Heights were measured to the nearest millimetre with a Seca plastic height board [29]. Weights were taken to the nearest tenth of a kilogram using a Camry model EB1003 scale [29]. Body mass index (BMI) of 30+ kg/m^2^ was categorized as having obesity class II, using Asian criteria [30].

*Tobacco use *was assessed with two questions: (1) “Have you ever chewed tobacco, smoked a pipe, smoked self-enrolled cigarettes, or smoked cigarettes/cigars?” (Yes, No); (2) “Do you still have the habit or have you totally quit?” (Still have, Quit) [29]. Responses were grouped into never, quitters and current tobacco users.

*Physical activity* was assessed with an abbreviated version of the “International Physical Activity Questionnaire (IPAQ) short version, for the last 7 days (IPAQ-S7S)” [31]. Physical activity was categorized according to the IPAQ scoring protocol [32] as low, moderate, and high physical activity.

*Nutrition risk* was assessed with the question “Concerning your food consumption, which of the following is true? 1=It is less than adequate for my needs, 2=It is just adequate for my needs, and 3=It is more than adequate for my needs.” (Coded 1=1, 2-3=0)

*Chronic medical conditions* were assessed with the question “Has a doctor/paramedic/nurse/midwife ever told you that you had…?” (“hypertension, diabetes or high blood sugar, heart attack, coronary heart disease, angina or other heart problems, stroke, tuberculosis, asthma, other lung conditions, liver, cancer or malignant tumor, arthritis/rheumatism, uric acid/gout, and depression”) (Yes, No) [29]. All chronic medical conditions were summed up to indicate if an individual had no, one, or two or more medical conditions

*Urinary problems* were measured with the question “Do you often get up during the night to urinate?” (Yes or No) [29].

*Vision and hearing problems* were assessed with the questions “Did a health care provider ever diagnose you with a vision problem, hearing problem?” “Do you/have you ever had a cataract?” (Yes or No) [29].

*Functional disability* was measured by ADL (5 items) and IADL (6 items) [33, 34]. ADL included the degree of having difficulty in performing dressing, eating, and other activities (Cronbach alpha of these five items was 0.84). Answers were categorized as “have no difficulty; have difficulty but can still do it; have difficulty and need help; cannot do it”. Responses were dichotomized into 1=one or more difficulties and 0=able, no difficulty. IADL included the degree of having difficulty in doing household chores, such as preparing meals and shopping (Cronbach alpha 0.91). A dichotomized functional disability total score was constructed and ADL/ IADL disability classified as having problems with in one or more ADL/IADL items.

*Depression symptoms* were assessed with the* Centres for Epidemiologic Studies Depression Scale (CES-D: *10 items), and score 15 or more was indicative severe depression symptoms [35] (Cronbach alpha 0.67).

*Sleep disturbance* was assessed with five items from the “Patient-Reported Outcomes Measurement Information System (PROMIS)” sleep disturbance measure [36]. A sample item was “I had difficulty falling a asleep.” Responses ranged from 1=not at all to 5= very much (Cronbach's alpha = 0.68). Sleep disturbance was defined as a score of three to five on the averaged mean items.

*Sleep related impairment* was assessed with five items from the PROMIS sleep impairment measure [37]. A sample item was “I had a hard time concentrating because of poor sleep.” Response options ranged from 1=not at all to 5= very much. (Cronbach's alpha = 0.82). Sleep related impairment was defined as a score of three to five on the averaged mean items.

*The balance test *(full tandem stand) was conducted according to standardized procedures [29], coded with <10 seconds or no attempt=1 and 10+ seconds=0.

*Hand grip strength *was estimated using a “Baseline Smedley Spring type dynamometer”, on each hand twice, beginning with the dominant hand, alternating hands in between measurements [29]. A maximum grip strength (kg) variable was created from all four measurements. Weak handgrip was classified as <20 kg for women and <30kg for men [38].

*Cognitive functioning* was assessed with questions from the Telephone Survey of Cognitive Status (TICS) [39], which was administered in a face-to-face interview in this study. The TICS included awareness of the date and day of the week and a self-reported memory question, with response options of excellent, very good, good, fair, and poor. Then the respondent was asked to serially subtract 7s from 100. Then an immediate and delayed word recall of 10 nouns was given [29]. Total scores ranged from 0-34; a score of 13 or lower was considered low.

### 2.3. Data Analysis

Descriptive statistics were calculated to describe the sample and occurrence of fall injuries.

Multinomial logistic regression analysis was computed to calculate the relative risk ratios (RRR) with 95% confidence interval (CI) to determine the associations between sociodemographic and health variables and single fall injury and multiple fall injuries, with no fall injury in the past two years as reference category. Associations between predictor variables and multiple fall injuries (with no fall injury as reference) were evaluated calculating odds ratios (OR) using unconditional multivariable logistic regression. All variables statistically significant at the p < .05 level in bivariate analyses were included in the multivariable models. Potential multicollinearity between variables was assessed with variance inflation factors, none of which exceeded critical value.* P* < 0.05 was considered significant. “Cross-section analysis weights were applied to correct both for sample attrition from 1993 to 2014 and then to correct for the fact that the IFLS1 sample design included oversampling in urban areas and off Java. The cross-section weights are matched to the 2014 Indonesian population, again in the 13 IFLS provinces, in order to make the attrition-adjusted IFLS sample representative of the 2014 Indonesian population in those provinces.” [29]. Both the 95% confidence intervals and P-values were adjusted considering the survey design of the study. All analyses were done with STATA software version 13.0 (Stata Corporation, College Station, TX, USA).

## 3. Results

### 3.1. Sample Characteristics and Prevalence Rate of Fall-Related Injury

The total sample included 6698 adults, 50 years and older (median age 58.0 years, IQR=11.0, and age range of 50-101 years) in Indonesia. The proportion of women was 51.9%, 72.2% had no or elementary education, 42.4% described themselves as having medium economic status, and 52.1% resided in urban areas. Regarding health variables, 18.0% of the participants reported nutrition risk, 7.4% measured having obesity, 48.4% had one more chronic condition, 56.2% had urinary problems, 1.3% had vision problems, 6.6% had or ever had a cataract, 3.4% had hearing problems, and 24.9% had one more functional disability. Almost one in five (17%) had depression symptoms, 14.7% sleep disturbance, 14.1% sleep impairment, 1.7% balance problems, 29.3% low cognitive functioning, and 61.5% weak hand grip strength.

Overall, 12.8% had one or more fall-related injuries in the past two years, 14.0% among women and 11.5% among men; 7.6% had a single fall and 5.2% multiple fall-related injuries in the past two years (see Table 1).

### 3.2. Associations with Fall-Related Injury

In adjusted analysis among both men and women, having two or more chronic conditions, urinary problems, and functional disability was associated with multiple fall-related injuries in the past two years. In addition, among men, former tobacco use, having or having had a cataract, sleep disturbance, and sleep impairment and, among women, poorer economic background, depression symptoms, and low cognitive functioning were associated with multiple fall-related injuries in the past two years (see Table 2).

## 4. Discussion

The study aimed to investigate the prevalence and health correlates of fall-related injury in a national sample of older Indonesians in 2014-15. A significant proportion of older adults in Indonesia had had a single and multiple fall-related injury, probably similar to previous studies in the region, e.g., China, India [4], Singapore [5], and Thailand [6]. Increasing age is a significant risk factor for fall-related injuries [5, 10–13]. In our study of adults 50 years and older, in unadjusted analysis, older age was not associated with a single fall injury but with multiple falls injury in the past two years, while the effect of older age disappeared in the fully adjusted models for both sexes. Possible reasons for this are under-reporting of fall injuries in the older age groups; only 5.5% of our sample was 80 years and older, due to higher fall-related mortality in the older age groups [4].

In agreement with previous studies [4, 5, 14], this study found that women were more likely than men to have any fall-related injury, in particular multiple falls. This gender disparity may be due to differences in higher levels of physical activity, muscle strength, bone density, and fatal fall rates in men than in women [40]. Although some studies found an association between lower socioeconomic status [14] and residing in rural areas [4, 15], this study only found an association between lower economic status and multiple fall injuries in women. It is possible that women with a lower economic status have more inadequate housing and other environments more prone for fall injuries to happen [8]. A previous study [16] found an association between nutritional risk and fall injury, while this study only found such an association in bivariate analysis in women. It is possible that the single item measure of nutrition risk was imprecise.

Previous studies [13, 19] found evidence that deficits in balance and in hand grip strength are risk factors for falls among older adults, while in this study only in crude analysis weak grip strength was associated with multiple fall injuries in men and balance problems in women. Moreland et al. [41] found in a systematic review of prospective cohort studies among older adults 65 years and above muscle strength, especially lower extremity muscles, was a significant risk factor for falls. Future studies should assess lower extremity muscles [13].

In agreement with previous evidence [4, 11–13, 16, 20, 21], this study found a dose-response relationship between the number of chronic conditions, functional disabilities, and fall-related injury. Having an increasing number of chronic conditions may negatively impact on one's mobility contributing to a higher fall risk. Urinary incontinence is a known risk factor for fall injury [13, 14, 18, 42], and we also found an association between urinary problems and multiple falls. Our measure of urinary problems consisted only of one item and the response option yes or no. This did not allow assessing the severity and type of urinary problems and have led to the overly high prevalence rate. Future studies should assess urge urinary incontinence, which was found in a systematic review to be associated with falls [42]. It is possible that urge urinary incontinence may lead to a loss of balance when rushing to the toilet or else urinary incontinence is a maker of frailty that is associated with higher fall risk [43].

Visual difficulties [14, 18], having cataracts [12], and hearing problems [23, 24] have previously been found risk factors for falls, while in this study only having cataracts was associated with multiple falls among men, and hearing problems were only significant in bivariate analysis in both sexes. Vision problems and/or having cataracts may increase the risk for falling because of obstacle avoidance based on diminished perception of spatial relationships and distances [16, 44].

Poor cognitive functioning has been found a risk factor for falls [4, 14], while in this study this was only found among women. Sleep problems and depression may be common in older people and there is evidence of an increased fall risk [4, 13, 28]. This study found that among men sleep disturbance and sleep impairment and among women depression symptoms were associated with single and multiple falls. Some researchers [45, 46] argue that “functional decline, history of falls, and cognitive impairment have been separately linked to both depression and fall.”

While most studies [5, 14, 18, 24, 25] found a protective effect of physical activity or exercise from fall injuries, this study found among men that high physical activity was associated with an increased risk of multiple falls. This may be partially explained “by reported changes in postural control among older men following lower or moderate physical activity that may be related to fatigue levels [43].”

Although obesity has been found a risk for falls in some studies in high income countries [12, 27], this study only found such an association in bivariate analysis among women. Some studies found an association between tobacco use and falls risk [26], while in this study an association between former tobacco use and falls was found among men. It is possible that former tobacco users had stopped the habit because of chronic diseases and increasing functional decline, which may explain why this group is at greater risk for fall injuries.

## 5. Limitations of the Study

This study had several limitations. The self-reported assessment of most study measures may have its limitations. Recall bias of two years fall injury and survivor bias may limit the robustness of the findings. Furthermore, this study was based on cross-sectional data, and we can therefore not ascribe causality to any of the associated factors in the study. Circumstances of falls and consequences in terms of type of injury were not assessed and should be assessed in future studies.

## 6. Conclusions

This study showed that a significant proportion of older adults in a national population-based survey in Indonesia had fall-related injuries in the past two years. Several homogenous between the sexes (multimorbidity, functional disability, and urinary problems) and sex-specific risk factors (sleep disturbance, sleep impairment, having cataracts and former tobacco use in men and depression and poor cognitive functioning in women) for fall-related injury were identified that can help in designing fall-prevention strategies.

## Figures and Tables

**Table 1:** Sample characteristics.

**Characteristic**	Sample	No Falls	Single fall	Multiple falls	P-Value
	%	%	%	%	
All	6698	5897 (87.2)	502 (7.6)	299 (5.2)	
Sex					
Male	3145 (48.1)	2786 (88.5)	233 (7.3)	126 (4.3)	<0.001
Female	3553 (51.9)	3111 (86.0)	269 (7.9)	173 (6.1)	
Age in years					
50-59	3772 (52.6)	3325 (88.2)	289 (7.6)	158 (4.2)	<0.001
60-69	1964 (27.9)	1725 (87.4)	143 (7.5)	96 (5.1)	
70-79	820 (14.0)	721 (85.3)	62 (7.6)	37 (7.2)	
80+	142 (5.5)	126 (81.1)	8 (8.2)	8 (10.7)	
Education (High school +)	2087 (27.8)	1811 (86.4)	194 (9.3)	82 (4.3)	<0.001
Economic background					
Poor	2070 (31.0)	1808 (87.4)	153 (7.6)	109 (5.0)	0.347
Medium	2842 (42.4)	2511 (88.4)	215 (7.5)	116 (4.1)	
Rich	1786 (26.7)	1578 (88.6)	134 (7.0)	74 (4.3)	
Residence (Urban)	3738 (52.1)	3260 (86.2)	316 (8.9)	162 (4.9)	<0.001
Body mass index (obese)	526 (7.4)	443 (84.1)	52 (9.2)	31 (6.7)	0.003
Tobacco use status					
Never	3895 (56.9)	3430 (87.0)	289 (7.5)	176 (5.5)	<0.001
Former	634 (9.8)	546 (84.3)	54 (8.9)	34 (6.8)	
Current	2169 (33.3)	1921 (88.3)	159 (7.3)	89 (4.3)	
Physical activity					
Low	3079 (44.3)	2713 (88.5)	234 (7.3)	132 (4.2)	0.663
Moderate	1880 (27.7)	1656 (88.0)	139 (7.6)	85 (4.4)	
High	1739 (28.1)	1528 (87.7)	129 (7.4)	82 (4.9)	
Nutritional risk (yes)	1259 (18.0)	1100 (87.0)	86 (6.9)	73 (6.1)	<0.001
Chronic conditions					
None	3451 (51.6)	3132 (90.3)	212 (6.3)	107 (3.4)	<0.001
One	1834 (27.4)	1620 (87.2)	143 (8.4)	71 (4.4)	
Two or more	1413 (21.0)	1145 (79.6)	147 (9.8)	121 (10.6)	
Urinary problems	3775 (56.2)	3262 (85.3)	297 (8.0)	216 (6.8)	<0.001
Vision problem	75 (1.3)	65 (86.2)	6 (7.5)	4 (6.3)	0.828
Cataract	451 (6.6)	367 (79.9)	46 (11.3)	38 (8.8)	<0.001
Hearing problem	218 (3.4)	182 (81.5)	16 (8.8)	20 (9.7)	<0.001
Functional disability					
ADL & IADL=0	4955 (75.1)	4423 (89.1)	344 (7.0)	188 (3.9)	<0.001
ADL & IADL=1	1315 (19.5)	1125 (86.2)	119 (8.6)	71 (5.2)	
ADL & IADL=2 or more	428 (5.5)	349 (81.5)	39 (8.7)	40 (9.8)	
Depression symptoms	1166 (17.0)	966 82.9	109 9.2	91 7.8	<0.001
Sleep disturbance (3-5)	1050 (14.7)	864 (82.6)	101 (9.4)	85 (8.0)	<0.001
Sleep impairment (3-5)	974 (14.1)	839 (85.5)	70 (7.1)	65 (7.4)	<0.001
Balance (no/<10s)	79 (1.7)	69 (85.0)	6 (7.0)	4 (8.0)	0.082
Cognitive function (low)	1794 (29.3)	1558 (88.0)	129 (6.5)	107 (5.5)	<0.001
Grip strength (weak)	4027 (61.5)	3546 (87.9)	290 (7.5)	190 (5.0)	<0.015

**Table 2:** Associations between sociodemographic, health variables and single and multiple fall-related injury.

**Characteristic**	**Men**			**Women**		
	Single fall	Multiple falls	Multiple falls	Single fall	Multiple falls	Multiple falls
	RRR (95% CI)	RRR (95% CI)	AOR (95% CI)	RRR (95% CI)	RRR (95% CI)	AOR
Age in years						
50-59	1 (Reference)	1 (Reference)	1 (Reference)	1 (Reference)	1 (Reference)	1 (Reference)
60-69	0.94 (0.75, 1.17)	1.30 (0.97, 1.75)	1.07 (0.76, 1.49)	1.06 (0.85, 1.31)	1.16 (0.90, 1.50)	0.89 (0.62, 1.28)
70-79	0.74 (0.53, 1.03)	1.85 (1.31, 2.63)*∗∗∗*	1.15 (0.71, 1.86)	1.26 (0.98, 1.63)	1.65 (1.25, 2.19)*∗∗∗*	1.01 (0.53, 1.95)
80+	0.86 (0.52, 1.42)	2.21 (1.35, 3.62)*∗∗*	1.26 (0.49, 3.24)	1.42 (0.99, 2.04)	2.93 (2.10, 4.08)*∗∗∗*	2.20 (0.48, 10.14)
Education (High school +)	1.25 (1.03, 1.53)*∗*	0.83 (0.63, 1.08)	-* *-* *-	1.60 (1.30, 1.96)*∗∗∗*	0.86 (0.66, 1.12)	-* *-* *-
Economic background						
Poor	1 (Reference)	1 (Reference)	-* *-* *-	1 (Reference)	1 (Reference)	1 (Reference)
Medium	1.09 (0.85, 1.38)	1.03 (0.75, 1.42)		0.86 (0.67, 1.09)	0.66 (0.49, 0.89)*∗∗∗*	0.66 (0.46, 0.96)*∗*
Rich	0.79 (0.58, 1.06)	0.86 (0.58. 1.26)		1.00 (0.77, 1.29)	0.83 (0.61, 1.13)	0.88 (0.59, 1.31)
Residence (Urban)	1.20 (0.99, 1.46)	0.72 (0.56, 0.92)*∗∗*	0.81 (0.60, 1.09)	1.73 (1.43, 2.09)*∗∗∗*	1.09 (0.89, 1.34)	-* *-* *-
Body mass index (obese)	1.48 (0.93, 2.36)	1.16 (0.60, 2.24)	-* *-* *-	1.21 (0.90, 1.61)	1.43 (1.04, 1.97)*∗*	1.37 (0.90, 2.06)
Tobacco use status						
Never	1 (Reference)	1 (Reference)	1 (Reference)	1 (Reference)	1 (Reference)	1 (Reference)
Former	1.57 (1.14, 2.16)*∗∗*	1.71 (1.15, 2.56)*∗∗*	1.77 (1.07, 2.92)*∗*	1.12 (0.65, 1.94)	2.40 (1.53, 3.77)*∗∗∗*	0.46 (0.10, 2.18)
Current	1.20 (0.92, 1.56)	1.19 (0.85, 1.68)	1.47 (0.96, 2.26)	1.18 (0.84, 1.67)	1.03 (0.68, 1.56)	0.88 (0.43, 1.83)
Physical activity						
Low	1 (Reference)	1 (Reference)	1 (Reference)	1 (Reference)	1 (Reference)	-* *-* *-
Moderate	1.21 (0.93, 1.58)	0.76 (0.50, 1.13)	0.78 (0.51, 1.17)	0.91 (0.72, 1.16)	1.26 (0.95, 1.66)	
High	1.12 (0.88, 1.44)	1.52 (1.11, 2.06)*∗∗∗*	1.49 (1.08, 2.07)	0.94 (0.72, 1.22)	0.88 (0.63, 1.25)	
Nutritional risk (yes)	1.04 (0.79, 1.37)	1.38, 0.99, 1.92)	-* *-* *-	0.85 (0.64, 1.12)	1.59 (1.20, 2.16)*∗∗∗*	1.28 (0.85, 1.92)
Chronic conditions						
None	1 (Reference)	1 (Reference)	1 (Reference)	1 (Reference)	1 (Reference)	1 (Reference)
One	1.41 (1.12, 1.77)*∗∗*	1.38 (1.01, 1.89)*∗*	1.28 (0.90, 1.83)	1.35 (1.08, 1.68)*∗∗*	1.28 (0.96, 1.71)	1.41 (0.91, 2.16)
Two or more	1.46 (1.13, 1.88)*∗∗*	2.56 (1.90, 3.73)*∗∗∗*	1.76 (1.19, 2.60)*∗∗*	1.96 (1.58, 2.45)*∗∗∗*	4.05 (3.17, 5.18)*∗∗∗*	3.67 (2.46, 5.45)*∗∗∗*
Urinary problems	1.22 (0.99, 1.48)	2.22 (1.67, 2.94)*∗∗∗*	1.58 (1.14, 2.19)*∗∗*	1.15 (0.96, 1.38)	2.19 (1.75, 2.76)*∗∗∗*	1.78 (1.26, 2.51)*∗∗∗*
Vision problem	0.64 (0.25, 1.61)	1.34 (0.58, 3.12)	-* *-* *-	1.65 (0.74, 3.68)	1.13 (0.29, 3.30)	-* *-* *-
Cataract	1.53 (1.05, 2.2`)*∗*	2.83 (1.95, 4.13)*∗∗∗*	2.02 (1.28, 3.19)*∗∗*	1.77 (1.32, 2.37)*∗∗∗*	1.43 (1.00, 2.03)*∗*	1.14 (0.65, 1.99)
Hearing problem	1.25 (0.76, 2.07)	2.06 (1.22, 3.47)*∗∗*	1.35 (0.73, 2.51)	1.25 (0.78, 2.01)	2.05 (1.32, 3.18)*∗∗∗*	1.66 (0.87, 3.17)
Functional disability						
ADL & IADL=0	1 (Reference)	1 (Reference)	1 (Reference)	1 (Reference)	1 (Reference)	1 (Reference)
ADL & IADL=1	1.48 (1.17, 1.88)*∗∗∗*	1.44 (1.04, 2.01)*∗*	1.14 (0.81, 1.61)	1.06 (0.82, 1.39)	1.37 (1.01, 1.88)*∗*	0.99 (0.70, 1.39)
ADL & IADL=2 or more	1.31 (0.81, 2.11)	3.33 (2.13, 5.20)*∗∗∗*	2.36 (1.43, 3.90)*∗∗∗*	1.41 (0.95, 2.08)	2.32 (1.55, 3.49)*∗∗∗*	1.65 (1.05, 2.60)*∗*
Depression symptoms	1.61 (1.24, 2.08)*∗∗∗*	2.22 (1.62, 3.03)*∗∗∗*	1.21 (0.84, 1.76)	1.24 (0.96, 1.60)	2.23 (1.70, 2.93)*∗∗∗*	1.80 (1.25, 2.59)*∗∗*
Sleep disturbance (3-5)	1.43 (1.11, 1.84)*∗∗*	2.06 (1.55, 2.75)*∗∗∗*	1.53 (1.06, 2.21)*∗*	1.44 (1.08, 1.91)*∗*	2.45 (1.78, 3.39)*∗∗∗*	1.15 (0.78, 1.69)
Sleep impairment (3-5)	0.83 (0.62, 1.12)	1.47 (1.08, 2.00)*∗*	1.95 (1.35, 2.83)*∗∗∗*	1.22 (0.90, 1.65)	2.67 (1.93, 3.68)*∗∗∗*	0.94 (0.63, 1.41)
Balance (no/<10s)	1.73 (0.76, 3.95)	0.71 (0.14, 3.58)	-* *-* *-	0.66 (0.30, 1.49)	1.99 (1.10, 3.57)*∗*	0.95 (0.29, 3.12)
Grip strength (weak)	1.08 (0.92, 1.35)	1.44 (1.08, 1.93)*∗*	1.14 (0.83, 1.58)	0.86 (0.69, 1.07)	1.22 (0.92, 1.61)	-* *-* *-
Cognitive function (low)	0.88 (0.68, 1.13)	1.24 (0.91, 1.70)	-* *-* *-	0.71 (0.55, 0.92)*∗∗*	1.34 (1.00, 1.79)*∗*	1.40 (1.00, 1.95)*∗*

RRR=relative risk ratio; AOR=adjusted odds ratio; *∗∗∗*P<0.001; *∗∗*P<0.01; *∗*P<0.05.

## Data Availability

The data underlying this study belong to the Indonesia Family Life Survey and are accessible via the RAND website http://www.rand.org/labor/FLS/IFLS.html. The authors did not have special access privilege.

## Additional Points

*Policy Impact. *Fall-related injuries were found to affect a significant proportion of adults 50 years and older in Indonesia. This representative community-based survey identified multiple risk factors that increase the likelihood of individuals having a single or multiple falls.* Practice Impact*. Agencies focusing on community-based fall prevention programmes should practice an integrated approach taking into account several homogenous between the sexes and sex-specific risk factors for fall-related injury.
